# How mRNA is misspliced in acute myelogenous leukemia (AML)?

**DOI:** 10.18632/oncotarget.2304

**Published:** 2014-11-08

**Authors:** Aminetou Mint Mohamed, Morgan Thénoz, Françoise Solly, Marie Balsat, Franck Mortreux, Eric Wattel

**Affiliations:** Oncovirologie et Biothérapies, CNRS UMR5239, Faculté de Médecine Lyon Sud, Ecole Normale supérieure de Lyon, Hospices Civils de Lyon, Pierre Bénite, France.

## Abstract

Approximately one-third of expressed genes are misspliced in AML, opening the possibility that additional factors than splicing factor mutations might cause RNA missplicing in these diseases. AML cells harbor a constellation of epigenetic modifications and regularly express large amounts of WT1 transcripts. Histone acetylation/methylation and DNA CpG methylation favor either exon skipping or inclusion, mainly through interfering with RNA Pol II-mediated elongation. This can result either from the binding of various factors on Pol II or alternatively from the recruitment of DNA binding factors that create roadblocks to Pol II-induced elongation. WT1 exhibits pleiotropic effects on mRNA splicing, which mainly result from the binding properties of WT1 via its zinc fingers domains to DNA, RNA, and proteins. Through the repression of the kinase SRPK1, WT1 modifies the splicing of VEGF, which plays important roles in hematopoiesis and angiogenesis. At the protein level, WT1 interacts with the splicing factors U2AF2, WTAP, and RPM4. Therefore, AML cells appear to have acquired numerous properties known to interfere with mRNA splicing. The challenge is now to elucidate these links in order to trigger mRNA splicing at the therapeutic level.

## INTRODUCTION

**Figure 1:**
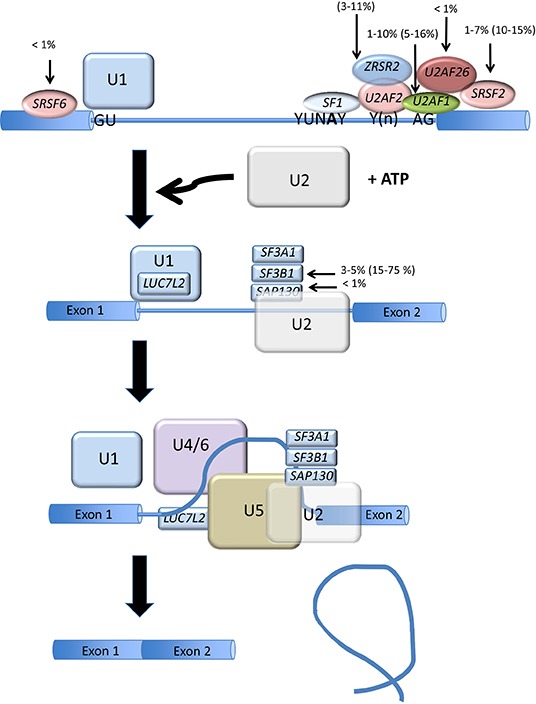
Splicing factor gene mutation in AML and MDS The two main steps of mRNA splicing are represented. The steps are catalyzed by spliceosomes and specified by three RNA sequence elements called the 5′ splice site, the 3′ splice site and the branch site (YUNAY). The 5′ splice site is recognized by the U1 snRNA-protein particle (snRNP) while the branch site is recognized by the U2 snRNP complexed with proteins at the 3′ splice site. In a later step of the spliceosome formation pathway, a tri-snRNP complex composed of U4, U5 and U6 snRNPs joins the spliceosome. Further rearrangements of the spliceosome lead to catalysis of the splicing reaction and the production of the spliced product mRNA and the excised intron. The main splicing factor gene mutation is represented for AML and MDS (in parenthesis).

Protein synthesis is a finely regulated process that begins with DNA replication, followed by transcription, and concluding with translation of the protein. Post-transcriptional modifications occur mainly between the transcription and translation processes, and ensure integrity and generate the diversity that characterizes the final protein products. Among post-transcriptional modifications, RNA splicing involves the removal of noncoding sequences (introns) from the primary transcript, or pre-mRNA (Figure 1). RNA splicing is orchestrated by small nuclear ribonucleoproteins (snRNPs), small nuclear RNAs, and protein factors that form spliceosomes. This process greatly expands the coding capacity of complex genomes, as it can generate protein products with distinct and even opposite properties from a single gene locus [1, 2]. It is therefore not surprising that dysfunction of alternative splicing (AS) can lead to various diseases, including cancers. Accordingly, abnormal AS has been demonstrated to contribute to many aspects of tumor initiation and addiction, including the control of cell proliferation and programmed cell death, metabolism of cancer cells, angiogenesis, metastasis, response to treatment and clinical outcome [3].

Acute myelogenous leukemia (AML) represents a heterogeneous spectrum of myeloid malignancies that harbor a constellation of chromosomal abnormalities, gene mutations, and epigenetic modifications. These genetic abnormalities have enabled understanding of the biology of the disease, helped generate the main diagnostic and prognostic tools, and represent key therapeutic targets. Recent reports have shown a significant deregulation of AS in AML, with approximately one-third of expressed genes being abnormally spliced in AML compared to normal CD34^+^ bone marrow cells [4]. Several reports have evidenced somatic mutations of splicing factors in AML, yet their frequency appears significantly lower than that observed in other myeloid malignancies such as in the myelodysplastic syndromes (MDS). In addition to spliceosome gene mutations, what other processes of factors can influence and alter AS in AML? Here we have concisely reviewed some of the main cellular processes known to influence AS, including those that are deregulated in AML such as deregulated transcription, epigenetic changes (histone modifications and CpG methylation), and WT1 gene overexpression.

### Abnormal mRNA splicing in AML and spliceosome gene mutations

Although abnormal RNA splicing has been previously demonstrated for several genes in AML, the connection between hematological malignancies and RNA splicing has recently emerged from studies based on next generation sequencing (NGS). Mutations in spliceosomal genes, in particular splicing factor 3 subunit b1 (SF3b1), were first identified in myelodysplastic syndromes (MDS) [5], myeloproliferative neoplasms (MPN) [6], MDS/MPN [7], and chronic lymphocytic leukemia (CLL) [8], as well as other hematological disorders [9]. Mutations in other spliceosomal genes such as U2 small nuclear RNA auxiliary factor 1 (U2AF1) [10] and serine arginine-rich splicing factor 2 (SRSF2) were subsequently identified [11]. The mutational landscape of the spliceosome is now available for hematological malignancies [12]. These findings have opened new avenues for understanding the underlying biology and for therapeutic intervention in this setting. Overall, the proportion of cases carrying spliceosome mutations ranges from <1–90% in MDS, MDS/MPN and secondary AML compared to <1–10.5% in de novo AML [5, 6] (Figure 1). In addition to this mutational spectrum, exon-array technologies and RNA sequencing (NGS) have enabled the assessment of the pattern of RNA splicing in hematological diseases. Certain spliceosome mutations have been found associated with missplicing in specific genes and might therefore help explain some aspects of the deregulated pattern of AS in AML. For example, in MDS, MPN, MDS/MPN, and secondary AML (sAML), U2AF1 (U2AF35) mutations, which are observed in 1–10% of de novo AML, have been found associated with abnormal splicing of genes involved in cell cycle progression and RNA processing that are somatically mutated or deleted in various cancers [13]. Yoshida et al. compared the effect of U2AF1 mutation on gene expression and splicing in HeLa cells and TF-1 myeloid cells [5]. The authors found that the S34F mutation quantitatively inhibited AS and triggered a significant enrichment of genes in nonsense-mediated mRNA decay (NMD), suggesting that the mutant U2AF35 triggered abnormal RNA splicing in HeLa and TF-1 cells, leading to the generation of unspliced RNA species carrying premature stop codons that induced NMD activity.

The development of natural compounds and synthetic analogues that target SFs is in progress [14]. The rationale for use of these spliceosome inhibitors in leukemia and MDS is that the majority of the splicing mutations found in hematological malignancies are heterozygous and considered gain of function or change of function/neomorphic mutations [5, 15]. Accordingly, it may be that treatment-triggered homozygous inactivation of spliceosomal genes may be more toxic to the heterozygous mutant cells than normal cells, and thus result in preferential killing of the mutant cells.

Recently Adamia *et al.* showed that approximately 29% of expressed genes are differentially and recurrently spliced in AML patients compared to normal bone marrow donors [4]. Alternative exon usages (AEUs) were found to involve oncogenes, tumor suppressor proteins, splicing factors and heterogeneous-nuclear-ribonucleoproteins, and proteins involved in apoptosis, cell proliferation, and spliceosome assembly [4]. *In silico*, these deregulations trigger important pathways involved in leukemogenesis. To date, their consequences on the physiopathology, diagnosis, response to treatment and disease outcome in AML remain unknown. In addition, little is understood regarding the causes and mechanisms that underlie abnormal AS in AML. Given such a proportion of abnormally spliced genes in AML, it could be proposed that additional factors other than spliceosomal mutations, which account for ≤10% of AML cases (Figure 1), would be involved in the deregulation of RNA splicing in AML. AS is influenced not only by signaling pathways that target the splicing machinery but also by transcription factors and chromatin structure. Accordingly, in addition to spliceosome gene mutations, numerous factors might influence RNA splicing in the context of AML. These include transcriptional deregulation, protein-protein interaction and epigenetic modifications.

### Epigenetics in alternative mRNA splicing

**Figure 2:**
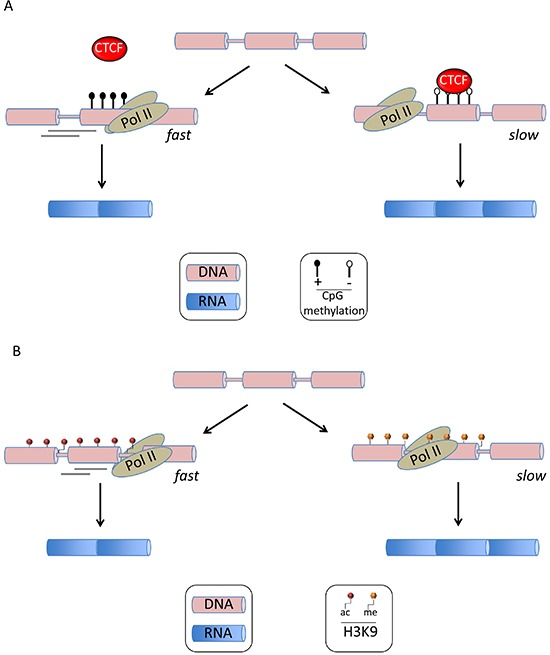
Epigenetic alterations in alternative mRNA splicing **(A)** DNA CpG methylation influences mRNA splicing. The figure illustrates the role of differential CpG methylation in the recruitment of cellular factors that create a roadblock to Pol-II-mediated elongation, resulting in stalling of transcription and favoring exon inclusion. This has been demonstrated by Shukla *et al.* with CTCF that binds unmethylated CG-rich DNA sequences located downstream of an alternative exon [20]. **(B)** Histone post-translational modifications influence mRNA splicing. Acetylation of histone 3 at Lys9 has been demonstrated by Schor *et al.* to increase Pol II-mediated elongation and thereby favoring exon skipping.

In addition to RNA sequence elements and their associated splicing factors, chromatin structure and histone modifications have been found to interfere with AS regulation (reviewed in ref [16]). This became apparent when mRNA splicing was found to be closely coupled with DNA transcription in mammalian cells [17]. In fact, transcription and splicing influence one another. For example, the transcription machinery can recruit several splicing factors such as SRSF3 that binds to the carboxy-terminal domain of RNA polymerase II (Pol II) [18]. Alternatively, some factors interacting with DNA may create roadblocks to Pol II-induced elongation [19]. Such pausing during elongation has been evidenced with the DNA-binding protein CCCTC-binding factor (CTCF) that promotes inclusion of an alternative exon 5 in CD45 through a roadblock effect [20] (Figure 2A).

The link between chromatin structure and splicing was first observed through the demonstration that the more compact chromatin structure of a replicated reporter plasmid acted as a barrier to Pol II-mediated elongation and resulted in higher exon inclusion [19]. Nucleosome positioning, histone modification and DNA methylation have been found to interfere with AS. Nucleosomes are preferentially positioned at exons and are thereby presupposed to create transient pauses to Pol II-mediated elongation, providing extra time for the recognition of 3′ splice sites by splicing factors [21]. Histone post-translational modifications represent a main regulator of AS (reviewed in ref [16, 19]) and acts through two distinct mechanisms. First, as illustrated in Figure 2B, some histone modifications, such as acetylation of histone 3 at Lys9 (H3K9ac), increase Pol II-mediated elongation and thereby favor exon skipping. Schor *et al.* demonstrated this for the neural cell adhesion molecule (NCAM) exon 18 upon neuron depolarization [19, 22]. Alternatively, the same study showed that neuron differentiation promoted inclusion of exon 18 in NCAM through H3K9 methylation (H3K9me), causing a reduction in Pol II-mediated elongation [19, 22]. Second, histone modifications can trigger the recruitment of factors influencing splicing, such as in the case of H3K36me3 at the fibroblast growth factor receptor 2 (FGFR2) locus that recruits the negative splicing factor PTB through the adaptor protein MRG15, resulting in exclusion of an alternative exon [23]. Abnormal DNA methylation represents a key pathogenic pathway in AML, yet its pathogenicity has mainly been linked to the global transcriptional deregulation of key genes involved in tumor development [24]. However recent studies have suggested that CpG methylation and transcriptional silencing are not synonymous [25]. Figure 2A gives a schematic example of how CpG differential methylation might influence AS [20]. Indeed, in the above example of CD45 splicing, Shukla *et al.* showed that DNA methylation of the intronic site prevented CTCF binding, releasing Pol II and thereby facilitating the skipping of exon 5 [20]. Epigenetic modifications are the hallmark of AML and several recent studies have highlighted the misregulation of DNA and histone methylation in this disease [25]. For example the role of abnormal DNA methylation in leukemia has recently been reinforced by the discovery of DNMT3A [26] and TET2 mutations [27] in about 20% of AML. DNMT3A is an active DNA methyltransferase [25]. Its gene mutations are regularly heterozygous and are predicted to disrupt the catalytic activity of the enzyme. TET2 converts 5mC into several oxidative intermediates, including 5-hydroxymethylcytosine (5hmC), which are likely involved in the process of active DNA demethylation. Similarly to DNMT3a substitutions in AML, patient-associated TET2 mutations are largely loss-of-function mutations that consequently result in decreased 5hmC levels and a reciprocal increase in 5mC. Regarding the deregulation of histone methylation, several histone lysine methyltransferases (KMT) have been found mutated in cancers. For example inactivation of EZH2 by loss or mutation are present in in MDS and, to a lesser extent in AML [25]. EZH2 is the catalytic component of the PRC2 complex, which is primarily responsible for the methylation of H3K27. ASXL1 is an additional epigenetic modifier found mutated in AML. ASXL1 mutations result in loss of polycomb repressive complex 2 (PRC2)-mediated histone H3K27 tri-methylation. Mutations in ASXL1 exon 12 are present in 5% - 30% of de novo AML where its frequency is 5 times higher in older patients [28], whereas some studies suggest a higher prevalence in secondary AML. Given the aforementioned link between missplicing and epigenetic changes, it will be therefore an important task to assess whether or not; missplicing is involved in the functional consequences of the mutation of epigenetic modifiers such as TET2, EZH2 and ASXL1 in AML.

### WT1 possesses pleiotropic effects on the splicing machinery

#### WT1 expression in AML

The Wilms' tumor gene (WT1) was among the first tumor suppressor genes to be cloned [29]. Originally named for its role in the pediatric kidney malignancy, Wilms' tumor, it has since been implicated in many other cancers including hematologic malignancies. In AML *ex vivo*, reduction of WT1 expression levels leads to decrease of proliferation and apoptosis of leukemic cells [30], indicating that WT1 acts as an oncogene in these diseases. Although the diagnostic level of WT1 expression does not seem to possess a significant prognostic impact, its decreasing level upon induction of chemotherapy has been found to correlate with subsequent favorable outcome. WT1 mutations occur in about 10% of AML with normal karyotype, whereas their prognostic impact remains unclear. In addition to its prognostic usefulness, WT1 has become a broadly used marker for minimal residual diseases and a promising therapeutic target for anti-sense molecules, antibodies, and vaccine strategies [31].

**Figure 3:**
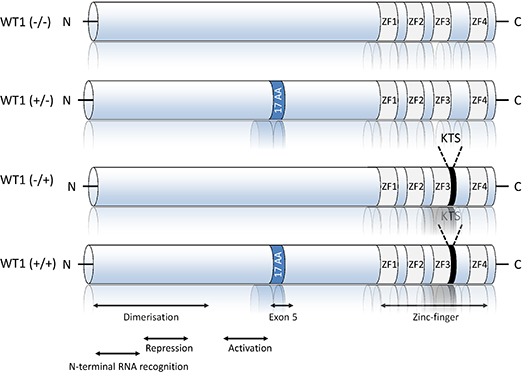
Schematic representation of the four main Wilms tumor suppressor gene (WT1) isoforms The best studied splice variants of WT1 include mammalian-specific 17 amino acids encoded by exon 5 and three amino acids (KTS) at the terminal end of zinc finger three, encoded by an alternative splice donor site in exon 9. These generate the four main WT1 isoforms, with molecular masses of 52–54 kDa. The N-terminal domain includes dimerization, transcriptional activation, RNA recognition [41, 66, 67] and repression domains. DNA and RNA binding activities are both mediated by the zinc-finger domain.

More than 30 different WT1 isoforms are generated by a combination of alternative RNA splicing, the usage of different start codons and RNA editing [32]. The most widely studied isoforms are the inclusion or exclusion of exon 5 and an alternative splice donor site in exon 9, which encodes three amino acids, KTS (Figure 3). Thus, WT1 can be exon 5+ or exon 5- and KTS+ or KTS-, and all four isoforms are expressed in several tissues [33]. Interestingly, some of these isoforms exhibit specific biological properties, and their expressions depend on the age of patients and disease phenotype (i.e. MDS vs. AML) [34, 35].

At the molecular level, WT1 is a zinc-finger DNA-binding protein that can act as a transcriptional activator or repressor depending on the cellular or chromosomal context [36] (Figure 3). As a zinc finger transcription factor, WT1 can bind DNA, RNA, and protein, affecting the flow of eukaryotic genetic information from transcription to translation. WT1 isoforms possess distinct biochemical properties. The -KTS isoform colocalizes in the nucleus with regions of active transcription and binds DNA, RNA, and protein, whereas the +KTS isoform is capable of binding to RNA and proteins and colocalizes with splicing machinery in nuclear speckles [37].

### Direct and indirect WT1-RNA interactions

The WT1-RNA interaction was first suggested by Larsson et al. who found that the +KTS WT1 isoform colocalizes with small nuclear RNA-protein particles (snRPs) in COS7 cells {Larsson, 1995 #148}. The mouse WT1 +KTS counterpart was further observed to accumulate on nascent transcripts when transfected in *Xenopus* oocyes [38], while Ladomery *et al.* [39] and Morrison *et al.* [40] identified the presence of WT1 in messenger ribonucleoprotein particles.

**Figure 4:**
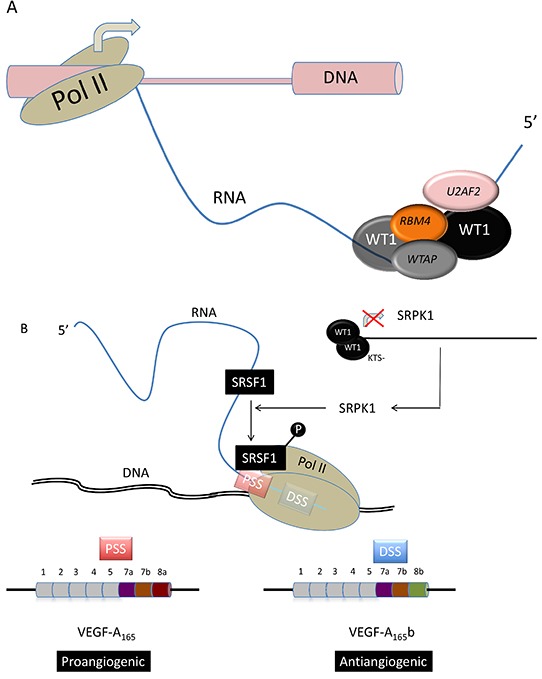
WT1 in alternative mRNA splicing **(A)** WT1 interacts with U2AF2, RBM4, and WTAP splicing factors. **(B)** –KTS (Figure 3) WT1 represses SRPK1 transcription, leading to SRSF1 hypophosphorylation and thereby for the selection of a 3′ proximal splicing site (PSS) during VEGF-A synthesis. This PSS usage results in the expression of the VEGF-A165 pro-angiogenic factor [52, 57].

Yeast two-hybrid screens identified the splice factors U2AF2 (U2AF65) [41], WTAP [42], and RBM4 [42] as WT1-interacting proteins (Figure 4A). U2 auxiliary factor 2 (U2AF2/65) interacts with the zinc finger domain of both + and –KTS WT1 isoforms via a serine-arginine domain within its N terminus. WT1 associated protein (WTAP) bounds the +KTS but not -KTS WT1 isoform (Figure 2A), and is involved in 3′ splice site selection [41]. Interestingly WTAP has been recently found to play an important role in abnormal proliferation and arrested differentiation of leukemia cells [44]. RNA-binding motif protein 4 (RBM4) is a splice factor that promotes skeletal muscle-specific exons [45] and is involved in stem-cell differentiation in the central nervous system [46] and the pancreas [47]. It is also involved in embryonic development in *Drosophila* and translational control in mouse and human [48, 49]. Markus *et al.* identified RBM4 as a binding partner of WT1 through yeast two-hybrid assays [43 d]. In contrast to U2AF65 and similar to WTAP, RBM4 is specific to the +KTS WT1 isoform [43] (Figure 2A). Mechanistically, minigene experiments have shown that WT1 counteracted the splicing effect of RBM4. Morrison *et al.* found that WT1 cofractionated and co-immunoprecipitated with the splice factor PSF in nuclear extracts prepared from mouse mesonephric fetal kidney M15 cells [50].

Besides its interaction with splice factors, WT1 interacts with RNA processing through RNA binding via its zinc finger domain [38, 51]. Accordingly WT1 is not restricted to the nucleus but shuttles between the nucleus and cytoplasm, where it is involved in the regulation of mRNA export, localization, and translation. *In vitro* binding assays have shown that a hairpin loop is critically required for RNA binding by WT1 zinc fingers [38]. Interestingly Morrison *et al.* found through immunoprecipitation coupled with PCR differential-display that WT1 interacted with the RNA of p54^nrb^, which encodes a splice factor related to PSF [50].

### Functional and pathogenic consequences of WT1-RNA interactions

The above-summarized interactions between WT1 and the splicing machinery are presupposed to alter the alternative exon usage that characterizes AML cells [4]. Two recent studies have addressed the consequences of WT1-RNA interactions and unmasked the effects of WT1 mutants on the splicing of the vascular endothelial growth factor (VEGF) and its consequences on angiogenesis, hematopoiesis, and tumor development [52, 53].

Angiogenesis is a key pathogenic mechanism in cancer and leukemia [54]. It is positively and negatively regulated by VEGF165 and VEGF165b splice isoforms, respectively [55]. VEGF splicing is controlled at least in part by serine-arginine-rich proteins (SRSFs), which include SRSF1, also called ASF, SF2, SF2/ASF, or ASF/SF2 [56]. SRSF1 promotes the expression of the angiogenic VEGF-A_165_ isoform via the selection of a specific 3′ proximal splicing site (3′ PSS) whereas the anti-angiogenic VEGF-A_165_b is expressed through the selection of a 3′ distal splicing site (3′ DSS, Figure 4B). Its nuclear localization is brought about by phosphorylation by a number of splicing factors, including SRPK1 [57], and Amin *et al.* demonstrated that WT1 isoforms lacking the KTS domain bind to the SRPK1 promoter and repress expression through a specific WT1 binding site [52] (Figure 4B). This repression results in SRSF1 hypophosphorylation that results in the selection of the 3′ DSS, thereby inhibiting angiogenesis through the expression of the VEGF-A_165_b isoform. In contrast, certain WT1 mutants carrying a substitution in the zinc-finger domain were unable to repress SRPK1 expression, leading to a SRPK1-mediated phosphorylation of SRSF1 that triggers the expression of the pro-angiogenic VEGF-A_165_ isoform [52].

In addition to their differential roles in angiogenesis, VEGF isoforms have been found to possess distinct effect on hematopoiesis. Cunningham *et al.* showed that WT1-deficient mouse embryonic stem cells exhibit reduced hematopoietic potential [53]. This diminished hematopoiesis was caused by a VEGF-A-dependent apoptosis of hematopoietic progenitor cells associated with a shift in VEGF-A isoforms toward VEGF-A 120. Interestingly, high levels of VEGF-121 (the human counterpart of murine VEGF-A120) have recently been identified as an independent prognostic factor associated with poor survival in AML [58].

### Conclusion and perspective

In addition to acquired and/or selected somatic mutations, AS offers AML cells a rapid, dynamic, and reversible means to deal with their environment, which depends on numerous factors influencing tumor development, maintenance and recurrence. These influencing factors include the bone marrow stroma, immune system and treatments. Given the huge excess of abnormally spliced genes in AML [4], it is reasonable to propose that alteration of splicing regulation participates in the phenotypic plasticity of AML cells. Accordingly, validating the *in silico* evidenced pathways deregulated in AML upon RNA missplicing will allow better assessment of the pathogenic implications of AS in this disease [4]. The example of S34F U2AF1 mutation, which has been demonstrated to trigger a specific pattern of AS [5], suggests that specific patterns of AS possessing specific functional consequences might be related to specific defects in the splicing process.

**Figure 5:**
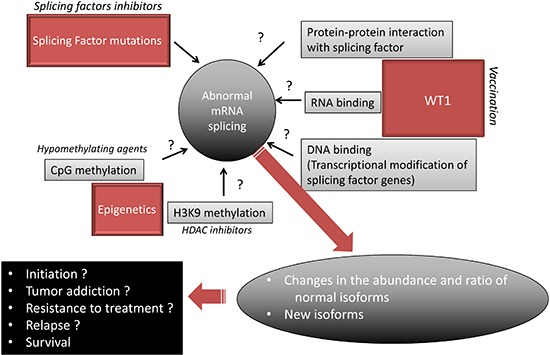
Proposed model of mRNA missplicing in AML Hypothetical mechanisms are denoted by a question mark. A targeted treatment is represented for each proposed mechanism.

Numerous questions remain to be addressed (Figure 5). For example, with the present reviewed data in mind, it becomes important to determine whether and how histone modifications, DNA methylation and WT1 expression modify AS and participate in the AML AS landscape. The main biological prognostic factors in AML have consisted of cytogenetic, gene mutations and transcriptional deregulations. Some patterns of AS have been found to correlate with tumor aggressiveness in solid tumors [58] while in AML, the expression of certain isoforms, such as for WT1 itself, TP53 [60], HOXA9 [61], BAALC [62], VEGF [58], or BCL-X [63], have been found to exhibit distinct effects on disease outcome. This encourages the investigation of the prognostic implication of the newly evidenced myriad of AML-associated alternative exon events [4]. Histone deacetylase inhibitors, DNA hypomethylating agents, and WT1 vaccination are currently used in many AML patients. Given that epigenetic and WT1 interfere with AS, it is possible that the antileukemic effect of these drugs relies at least in part on modification of AS. A recent meta analysis of published cancer vaccine trials has shown that objective clinical responses (including stable disease) are observed in 64% of evaluable WT1-vaccinated patients with hematological malignancies while immunogenicity of WT1-based cancer vaccines was demonstrated by the detection of a specific immunological response in 68% of cases [64]. Some drugs directly target the spliceosome, such as spliceostatin that targets SF3B1, have been found to possess antitumor effect ex vivo [14]. Whether these specific splicing factor inhibitors act in non-mutated AML remains to be assessed. In addition to these approachs, steric-blocking oligonucleotides have been designed to specifically redirect alternative splicing, repair defective RNA, and restore protein production. Although these product were designed to treat genetic disorders such as Duchenne muscular dystrophy [65], it will be interesting to develop such strategy to target the specific splicing defects observed in AML.
